# SINGLE-PORT FOR LAPAROSCOPIC GASTRIC RESECTION WITH A NOVEL PLATFORM

**DOI:** 10.1590/S0102-67202014000200015

**Published:** 2014

**Authors:** Marcel Autran MACHADO, Fabio F. MAKDISSI, Rodrigo C. SURJAN

**Affiliations:** 1 Department of Surgery, Sirio Libanes Hospital (1Departamento de Cirurgia, Hospital Sirio Libanes), São Paulo, SP, Brazil; 2 Department of Gastroenterology, University of São Paulo (2Departamento de Gastroenterologia, Universidade de São Paulo), São Paulo, SP, Brazil

**Keywords:** Gastrointestinal duplication, Single-port, Laparoscopy, Stomach, Technique

## Abstract

**Introduction:**

Laparoscopic gastrointestinal resections using single-port are possible, but triangulation problems and the need of articulated instruments difficult the procedures.

**Aim:**

To present a surgical alternative using single-port laparoscopic device on gastric resection.

**Technique:**

The patient is placed in a supine and reverse Trendelenburg position with surgeon between patient's legs. First assistant was on the right side of the patient with the monitor placed on the patient's cranial side. With the patient under general anesthesia, a transumbilical 3 cm skin incision is performed. A single-incision advanced access platform with gelatin cap, self-retaining sleeve and wound protector is introduced through this incision. Three 5-12 mm operating ports were introduced through the single-port device. Due to the gel cap and sleeves, no articulated instruments are necessary. CO_2_ pneumoperitoneum is established at 12 mmHg. A rigid 30 degree 10 mm laparoscope is introduced. Operation begins with access to the lesser sac by opening the omentum along the greater curvature of the stomach using harmonic scalpel. Once the stomach is fully exposed and a stay suture is place around the tumor. Gastric wall is divided with cautery 1 cm away from the tumor. Tumor is excised. Gastric wall is sutured with two-layer running suture. No drain was used. Umbilical incision was closed.

**Results:**

This procedure was used in one patient with gastric duplication. Operative time was 200 minutes. Blood loss was minimal. Recovery was uneventful and patient discharged on postoperative day 2. Final aspect of the umbilical incision was good.

**Conclusions:**

Gastric resection with single-port laparoscopic platform is feasible and may be safely performed in selected patients.

## Abstract (pt)

**Introdução:**

Ressecções gastrointestinais laparoscópicas com portal único são possíveis, mas os problemas de triangulação e a necessidade de instrumentos articulados dificultam a realização destes procedimentos.

**Objetivo:**

Apresentar uma alternativa cirúrgica utilizando dispositivo laparoscópico de portal único na ressecção gástrica.

**Técnica:**

O paciente é colocado em posição supina e em Trendelenburg reverso com o cirurgião entre as pernas. Auxiliar é posicionado ao lado direito do paciente com o monitor cranialmente situado. Sob anestesia geral, incisão transumbilical de 3 cm é realizada. Através dela, uma plataforma de portal único, com cobertura gelatinosa é introduzida. Três portais de trabalho de 5-12 mm são introduzidos através do dispositivo de portal único. Devido à cobertura gelatinosa, instrumentos articulados não são necessários. O pneumoperitônio é estabelecido em 12 mmHg. É utilizada ótica rígida de 30 graus com introdução de laparoscópio de 10 mm. A operação começa com abertura do omento maior ao longo da grande curvatura do estômago usando bisturi harmônico. Após exposição do estômago, pontos de reparos são colocados e gastrectomia parcial é realizada a cerca de 1 cm do tumor. O procedimento é completado pela sutura contínua da parede gástrica em dois planos. Não é necessária drenagem da cavidade e a incisão umbilical é suturada.

**Resultados:**

Este procedimento foi usado em um paciente com a duplicação gástrica. O tempo operatório foi de 200 min. A perda de sangue foi mínima. A recuperação transcorreu sem intercorrências e o paciente recebeu alta no 2º dia do pós-operatório. O aspecto final da incisão umbilical foi excelente.

**Conclusão:**

A ressecção gástrica laparoscópica com o uso de portal único é factível e pode ser empregada com segurança em pacientes selecionados.

## INTRODUCTION

In the past decade, minimal access surgery is moving towards minimizing the surgical trauma by reducing numbers and size of the portals. In the last few years, a novel technique with a single-incision laparoscopic approach has been described^3^. This technique has mainly been used for laparoscopic cholecystectomy, but recent reports showed feasibility even in more complex operations such as gastric, liver and pancreas resection^12,13,19^.

In the English literature, there are few papers dealing with single-port laparoscopic gastrointestinal resections. The main reason is that the majority of systems available for a single-port laparoscopic surgery needs specific articulating instruments, use of small laparoscopes and allows poor triangulation^3,19^. The use of a new single-port platform based on gelatin cap is essential to avoid triangulation problems and precluded the use of articulated instruments.

The objective of this paper is to present technical details of single-port laparoscopic partial gastrectomy and the use of this procedure in one patient with gastric duplication.

## TECHNIQUE

**Figure 1:**
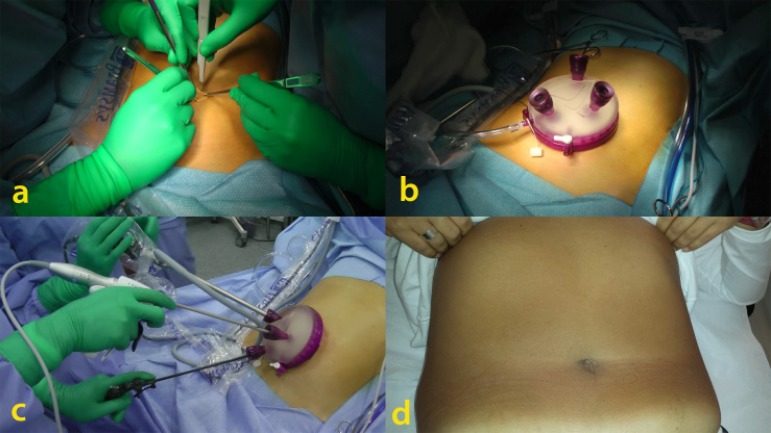
Single-port platform set-up: a) umbilical incision is performed; b) gelatin cap is attached to the platform with three working (5 to 12 mm) ports; c) the single-port is able to accommodate at the same time three instruments with no triangulation prejudice; d) final view of umbilical wound nine months after the procedure

**Figure 2:**
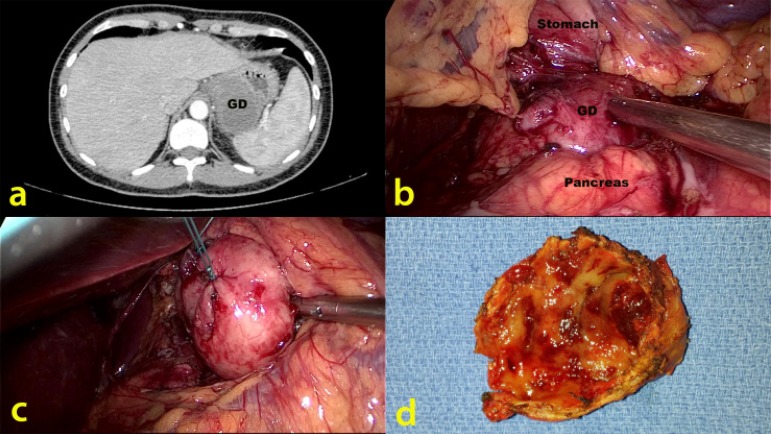
Single-port laparoscopic resection of gastric duplication: a) CT scan discloses a large cyst (GD=gastric duplication) behind stomach with close contact with splenic hilum and body of the pancreas; b) internal view shows a large cyst adjacent to the body of the pancreas but with a clear clivage plan, pancreas was separated from the cyst with blunt dissection where no cleavage plan existed with gastric wall and intraoperative diagnosis of gastric duplication was confirmed; c) internal view shows suture of gastric wall after resection of the gastric duplication; d) surgical specimen.

The patient is placed in a supine and reverse Trendelenburg position with surgeon between his legs. First assistant is on the right side with the monitor placed on the patient's cranial side. Under general anesthesia, a transumbilical 3 cm skin incision is performed (Figure 1a). A single-incision advanced access platform with gelatin cap, self-retaining sleeve and wound protector (GelPoint, Applied Med. R.S. Margarita, CA, USA) is introduced through this incision. Three 5-12 mm operating ports are introduced through the single-port device (Figure 1b). Due to the gel cap and sleeves, no articulated instruments are necessary. CO_2_ pneumoperitoneum is established at 12 mmHg. A rigid 30 degree 10 mm laparoscope is introduced. The single-port is able to accommodate at the same time three instruments with no triangulation prejudice: a 10 mm laparoscope, a 12 mm ultrasound probe and a 5 mm instrument such as harmonic scalpel, grasper, scissor or dissector (Figure 2c).

Operation begins with access to the lesser sac by opening the omentum along the greater curvature of the stomach using harmonic scalpel (Ultracision, Ethicon Endo Surgery, Cincinnati, OH, USA). Once the stomach is fully exposed a stay suture is place around the tumor. Gastric wall is divided with cautery 1 cm away from the tumor and the tumor is excised. Gastric wall is closed with running suture in one or two layers. No drain is used. Umbilical incision is closed (Figure 1d).

## RESULTS

This procedure was used in a 47-year-old woman with a 4.2 cm retroperitoneal cystic lesion found by routine ultrasonography. Endosonography^6^, MIR, CT and biopsy findings were consistent with pancreatic mucinous cystadenoma (Figure 2a). The initial plan was to perform a single-port distal pancreatectomy with splenic preservation. Intraoperative ultrasound (SonoSite, Inc., Bothell, WA, USA) indicated that the tumor was not from pancreatic origin. Absence of cleavage plane between gastric wall and the cystic tumor was consistent with the diagnosis of gastric duplication that was resected and removed through the single-port with the technic above described. Further dissection was done and it was able to dissect the cystic tumor away from the superior border of the pancreas. Absence of cleavage plane between gastric wall and the cystic tumor was consistent with the diagnosis of gastric duplication (Figure 2b). Gastric duplication was finally removed without opening of the cyst or gastric mucosa. Surgical specimen was removed through the single-port (Figure 2c and d). Gastric mucosa was closed with running suture. Umbilical incision was sutured and abdominal cavity was not drained. Frozen section confirmed the diagnosis and no malignancy were found in the surgical specimen.

Operative time was 200 minutes. Blood loss was minimal, and the patient did not receive transfusion. The recovery was uneventful, and the patient was discharged on postoperative day 2. Final pathology disclosed gastric duplication without malignant transformation. Patient had no signs of disease at nine months after operation. Final aspect of the umbilical incision was good (Figure 1d).

## DISCUSSION

To the best of knowledge of the authors, this is the first case of gastric duplication treated by this method reported in the English literature.

Gastrointestinal duplications are rare congenital malformations that may arise from the oral cavity to the rectum. The ileum is more frequently affected, while gastric duplications comprises as low as 4% of all digestive duplications. ^1,9^. Its usual location is the greater curvature^16,18,22^. Sixty-seven percent of all gastric duplication cysts are diagnosed in the first year of life, when they present abdominal mass and gastric obstruction. Less than 25% of the cases are diagnosed after 12 years of age and in adult life is very rare, but may be associated with malignant degeneration^4,5,7,10,11,15,22,23^. In this setting, diagnosis may be difficult, as gastric duplications are usually asymptomatic or present with vague symptoms, such as weight loss, anemia, epigastric fullness or nausea^8^. The author's previous experience with gastric duplication resection showed that gastric duplication usually shared the same seromuscular layer with the stomach. Therefore, meticulous dissection was needed in order not to open the gastric mucosa. However, whenever necessary, as in cases of more infiltrative neoplasms, opening of gastric mucosa can safely be performed.

The novel single-port platform may increase the adoption of single-port operations. Although several issues such as costs and learning curve of this technique remain to be studied, the cosmetic benefits of single-incision approach are obvious.

There are very few published articles on laparoscopic resection of duplication cysts in adults affecting the stomach^14,17,20,21^.

In the past decade, minimal access surgery is moving towards minimizing the surgical trauma by reducing numbers and size of the port. In the last few years, a novel technique with a single-incision laparoscopic approach has been described^3^. It is especially used in young female due to the good cosmetic results. Single-incision laparoscopic procedure is less invasive than standard multiport laparoscopy but may have unique difficulties for the laparoscopic surgeon^12,13^. First, retraction is significantly limited. The introduction of a camera and several instruments parallel to each other may result in decreased range of motion and collision of instruments^2^. The single-port platform used in this case allowed the use of standard instruments with no loss of triangulation due to the presence of self-retaining sleeves which maximizes internal working diameter. It was able to use a high definition 10 mm laparoscope during all steps of the operation.

## CONCLUSION

Single-port laparoscopic gastric resection is feasible. This new surgical platform may increase the adoption of single-port operations. Although several issues such as costs and learning curve of this technique remain to be studied, the cosmetic benefits of single-incision approach are obvious.
